# Supplementary material for: Solar cycle as a distinct line of evidence constraining Earth’s transient climate response
Source: Nat Commun. 2023 Dec 19;14:8430. doi: 10.1038/s41467-023-43583-7 (PMC10730699; doi:10.1038/s41467-023-43583-7)
Supplement: Supplementary file 1 — Supplementary Information [file 41467_2023_43583_MOESM1_ESM.pdf]

Supplementary Information
of

Solar Cycle as a Distinct Line of Evidence Constraining Earth's Transient Climate Response

King-Fai Li[#] and Ka-Kit Tung*
[#] Department of Environmental Sciences, University of California, Riverside
*Department of Applied Mathematics, University of Washington, Seattle
Corresponding author: ktung@uw.edu

Supplementary Text

This document provides the mathematical details of the Linear Discriminant Analysis (LDA) used in the main text to extract the solar responses in observational datasets and CMIP6 historical simulations (Supplementary Section S1). It also provides a list of CMIP6 models and their simulation configurations, such as the duration of the spin-up simulations and the transient climate sensitivities, which have been used in the main text (Supplementary Section S2).

S1. The Linear Discriminant Analysis (LDA)

S1.1. The general formulism

The LDA was first introduced by Fischer in 1936[1]. Let $\mathbf{Y}(t,x)$ be an $n \times p$ geophysical dataset that contains measurements at n timesteps and p locations. $\mathbf{Y}(t,x)$ is assumed to have centered in time, i.e., the temporal mean at each location is zero. Let $\mathbf{G}(t,g)$ be a group matrix for specifying g groups in time: If the i-th timestep belongs to the j-th group, then $\mathbf{G}(i,j)=1$; otherwise, $\mathbf{G}(i,j)=0$. $\mathbf{G}^T\mathbf{G}=\text{diag}(n_j)$ is a $g \times g$ diagonal matrix containing the number of elements in the j-th group. $(\mathbf{G}^T\mathbf{G})^{-1}\mathbf{G}^T$ is a within-group averaging operator, so that $\mathbf{M}=(\mathbf{G}^T\mathbf{G})^{-1}\mathbf{G}^T\mathbf{Y}$ is a $g \times p$ matrix of g group means at all locations. The $p \times p$ within-group covariance matrix is given by $\mathbf{\Sigma}_w=\frac{1}{n-g}(\mathbf{Y}-\mathbf{GM})^T(\mathbf{Y}-\mathbf{GM})$. The $p \times p$ between-group covariance matrix is given by $\mathbf{\Sigma}_b=\frac{1}{(g-1)}(\mathbf{GM})^T(\mathbf{GM})$. The $p \times p$ total covariance matrix is given by $\mathbf{\Sigma}_t=\frac{1}{(n-1)}[(n-g)\mathbf{\Sigma}_w+(g-1)\mathbf{\Sigma}_b]$.

The LDA aims to find a discriminant vector $\mathbf{a}$ that maximizes the ratio $\gamma=(\mathbf{a}^T\mathbf{\Sigma}_t\mathbf{a})^{-1}(\mathbf{a}^T\mathbf{\Sigma}_b\mathbf{a})$. Given the condition $\frac{\partial\gamma}{\partial\mathbf{a}}=0$, γ and $\mathbf{a}$ are the eigenvalues and eigenvectors of the matrix equation $\mathbf{\Sigma}_t^{-1}\,\mathbf{\Sigma}_b\mathbf{a}_k=\gamma_k\mathbf{a}_k$, where k denotes the k-th eigen component. $\mathbf{c}_k=\mathbf{X}\mathbf{a}_k$ is the $n \times 1$ projected time series corresponding to the k-th eigencomponent. $\mathbf{p}_k=(\mathbf{a}_k^T\mathbf{\Sigma}_t\mathbf{a}_k)^{-1}(\mathbf{\Sigma}_t\mathbf{a}_k)$ is the k-th $p \times 1$ discriminating spatial pattern, which is mathematically equivalent to the regression coefficients of $\mathbf{c}_k$ projected on $\mathbf{Y}$ at each location.

S1.2. The numerical algorithm

Supplementary Section S1.1 discusses the general principle of the linear discriminant analysis and the physical meaning of γ_k and $\mathbf{a}_k$. Here, we present the numerical algorithm we use to obtain γ_k and $\mathbf{a}_k$. This numerically stable and efficient algorithm is based on the discussion in Riley[2].

S1.2.1. Data reduction

Let $\frac{\mathbf{Y}}{\sqrt{n-1}} = \mathbf{U\Delta V}^T$ be the singular value decomposition (SVD) of $\mathbf{Y}$ divided by the degrees of freedom in time. A whitening matrix $\mathbf{S}$ is defined as the inverse of some "square root" of the total covariance matrix $\mathbf{\Sigma}_t$. Given the SVD of $\frac{\mathbf{Y}}{\sqrt{n-1}}$, we may take as $\mathbf{S} = \mathbf{V}_r\mathbf{\Delta}_r^{-1}$, where $\mathbf{V}_r$ and $\mathbf{\Delta}_r$ are the reduced $\mathbf{V}$ and the reduced $\mathbf{\Delta}$ containing only the first r singular components. r will be a truncation parameter that regularizes $\mathbf{Y}$; see Supplementary Section S1.3. The left inverse of $\mathbf{S}$ is given by $\mathbf{S}_L^{-1} = \mathbf{\Delta}_r\mathbf{V}_r^T$. The whitened dataset $\widetilde{\mathbf{Y}}$ is given by $\mathbf{YS}$, which is equivalent to $\sqrt{n-1}\mathbf{U}_r$, where $\mathbf{U}_r$ is the reduced $\mathbf{U}$. Thus, $\widetilde{\mathbf{Y}}$ is reduced to a $n \times r$ matrix.

In SVD, the truncation parameter r is usually taken to be the rank of the data matrix, i.e., singular components greater than the rank are constant components in the null space. However, such a rank does not necessarily regularize $\mathbf{Y}$ with respect to the process of interest (the 11-year solar cycle signal in our case). Before the LDA analysis of the global warming signal, Schneider and Held[3] proposed a linear regression approach $\mathbf{G} = \mathbf{Y\beta}$ ($\mathbf{\beta}$ being the regression coefficients) to pre-determine the truncation parameter using the generalized cross-validation. Their approach works if the physical phenomenon is the dominant variability in the data. However, for our 11-year solar cycle study, solar modulation is generally weaker than other natural variability. There, the Schneider-Held regularization likely returns $r = 1$, meaning that only the arithmetic mean of the data is retained and that the solar-cycle signal is removed from the regularization. Instead of the generalized cross-validation, we post-determine r based on the values of the extracted solar-cycle response κ, the correlation coefficient of the projected time series ($\mathbf{YSV}'$ in Supplementary Section S1.2.3) with the solar index, and the separation parameter γ. We will discuss the determination of r in Supplementary Section S1.3.

S1.2.2. The discriminants for $\widetilde{\mathbf{Y}}$

Since the total covariance of the whitened dataset is unity by construction, the discriminant is simply the eigenvector of the between-group covariance matrix $\widetilde{\mathbf{\Sigma}}_b = \frac{1}{g-1}\left(\mathbf{G}\widetilde{\mathbf{M}}\right)^T\left(\mathbf{G}\widetilde{\mathbf{M}}\right)$, where $\widetilde{\mathbf{M}} = (\mathbf{G}^T\mathbf{G})^{-1}\mathbf{G}^T\widetilde{\mathbf{Y}}$. One may perform an eigen-decomposition on $\widetilde{\mathbf{\Sigma}}_b$ directly but the eigen-decomposition is not numerically efficient. Instead, a more popular way is to perform an SVD on a square root of $\widetilde{\mathbf{\Sigma}}_b$. An obvious square root is proportional to $\frac{1}{\sqrt{g-1}}\mathbf{G}\widetilde{\mathbf{M}}$ but $\mathbf{G}\widetilde{\mathbf{M}}$ has the same dimension $n \times r$ as $\widetilde{\mathbf{Y}}$, which may be large if n is large. Instead, a square root of $\mathbf{G}^T\mathbf{G}$ is introduced. In particular, the $g \times g$ diagonal matrix $\mathbf{T} = \mathrm{diag}\left(\sqrt{n_j/n}\right)$ is a square root of $\mathbf{G}^T\mathbf{G}$ because $\mathbf{T}^{-1}\mathbf{G}^T\mathbf{G}\mathbf{T}^{-1} = n\mathbf{I}$. With $\mathbf{T}$, $\left(\mathbf{G}\widetilde{\mathbf{M}}\right)^T\left(\mathbf{G}\widetilde{\mathbf{M}}\right)$ may be rewritten as $n\left(\mathbf{T}\widetilde{\mathbf{M}}\right)^T\left(\mathbf{T}\widetilde{\mathbf{M}}\right)$. Thus, $\sqrt{\frac{n}{g-1}}\mathbf{T}\widetilde{\mathbf{M}}$ is a square root of $\widetilde{\mathbf{\Sigma}}_b$ with a smaller size $g \times r$, enabling a more efficient SVD processing. Let $\mathbf{T}\widetilde{\mathbf{M}} = \mathbf{U}'\mathbf{\Delta}'\mathbf{V}'^T$ be the SVD. Then $(g-1)\widetilde{\mathbf{\Sigma}}_b = n\,\mathbf{V}'\mathbf{\Delta}'^2\mathbf{V}'^T$, suggesting that $\mathbf{V}'$ contains the eigenvectors of $\widetilde{\mathbf{\Sigma}}_b$, which are the desired discriminants for $\widetilde{\mathbf{Y}}$.

[Incidentally, since $(n-g)\widetilde{\mathbf{\Sigma}}_w = \widetilde{\mathbf{Y}}^T\widetilde{\mathbf{Y}} - (g-1)\widetilde{\mathbf{\Sigma}}_b = n\mathbf{I} - n\mathbf{V}'\mathbf{\Delta}'^2\mathbf{V}'^T = n\mathbf{V}'(\mathbf{I} - \mathbf{\Delta}'^2)\mathbf{V}'^T$, $\mathbf{V}'$ is also an eigenvector of $\widetilde{\mathbf{\Sigma}}_w$.]

S1.2.3. The discriminants for $\mathbf{Y}$

Finally, $\mathbf{V}'$ needs to be transformed back to the original dataset. Since $\mathbf{\Sigma}_t = (\mathbf{SS}^T)^{-1}$ and $\mathbf{\Sigma}_b = \frac{n}{g-1}\mathbf{S}_L^{-T}\widetilde{\mathbf{M}}^T\mathbf{T}^2\widetilde{\mathbf{M}}\mathbf{S}_L^{-1}$, $\mathbf{\Sigma}_t^{-1}\,\mathbf{\Sigma}_b$ is proportional to $\mathbf{S}\widetilde{\mathbf{M}}^T\mathbf{T}^2\widetilde{\mathbf{M}}\mathbf{S}_L^{-1}$, which can be rewritten in the

diagonalized form $(\mathbf{SV}')\mathbf{\Delta}^2(\mathbf{SV}')^{-1}$. Thus, $\mathbf{SV}'$ are the eigenvectors of $\mathbf{\Sigma}_t^{-1}\mathbf{\Sigma}_b$ and hence the desired discriminants. The discriminating spatial patterns are given by $[(\mathbf{SV}')^T\mathbf{\Sigma}_t\mathbf{SV}']^{-1}(\mathbf{\Sigma}_t\mathbf{SV}')$. Since $(\mathbf{SV}')^T\mathbf{\Sigma}_t\mathbf{SV}'$ is an identity matrix, the discriminating spatial patterns are given by the columns of $\mathbf{\Sigma}_t\mathbf{SV}'$, which may be simplified as $\mathbf{S}_L^{-T}\mathbf{V}'$. The projected time series are given by the columns of $\mathbf{YSV}'$.

S1.2.4. The extracted solar response κ

To extract the solar-cycle response in the surface temperature, we use the total solar irradiance, $TSI(t)$, as the training index to define the values in $\mathbf{G}(t,g)$. If $TSI(t)$ is greater than its mean over the period 1950–2004, then $\mathbf{G}(t,1)$ is set to 1 and $\mathbf{G}(t,2)$ is set to 0. In contrast, if $TSI(t)$ is less than its mean over the period 1950–2004, then $\mathbf{G}(t,1)$ is set to 0 and $\mathbf{G}(t,2)$ is set to 1. The first discriminant is the solar-cycle response in the surface temperature. Therefore, the first row of $\mathbf{\Sigma}_t\mathbf{SV}'$ is the solar-cycle spatial pattern, and the first column of $\mathbf{YSV}'$ is the projected time series of the solar-cycle response.

The extracted solar-cycle response, κ, in the unit of °C/W m^{-2} is defined as the linear regression coefficient between the projected time series and $TSI(t)$.

S1.3. Choice of r

The truncation parameter r discussed in Supplementary Section S1.2.1 has not been determined so far. r can be any integer between 1 and the smaller value of n and p, i.e. $\min([n,p])$. We perform the LDA analysis for all possible values of r. For each r, a different $\widetilde{\mathbf{Y}}$, and hence a different projected time series, is obtained. Then we calculate three quantities: the extracted solar-cycle response κ, the correlation coefficient ρ with $TSI(t)$, and the separation parameter γ of the first discriminant. Supplementary Figure S1 shows an example using HadCRUT5. The maximum possible value of r is 58. As r increases from 2 to 58, ρ and γ generally increase slowly except at

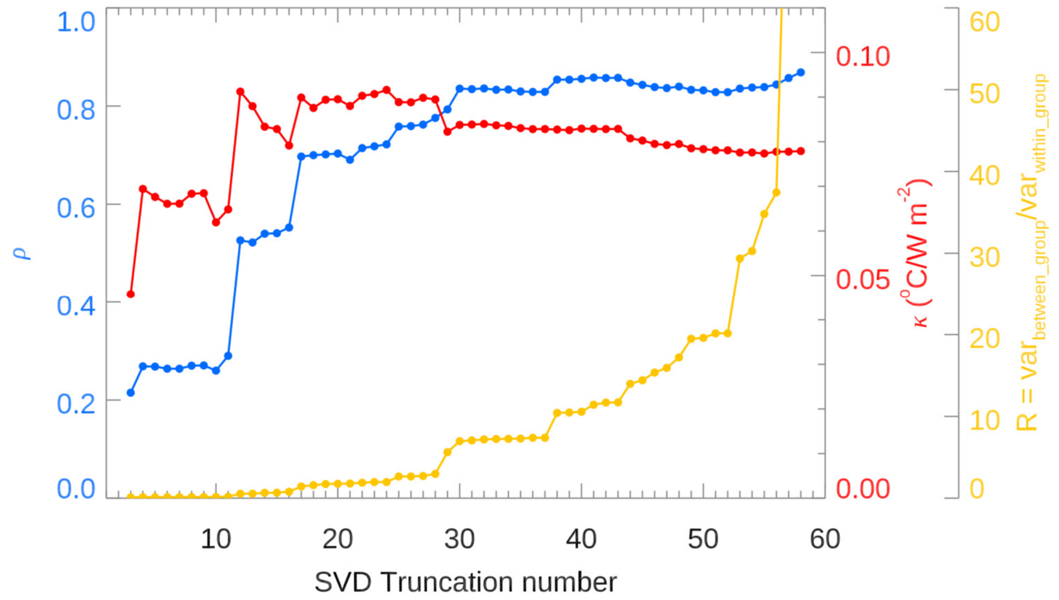

Supplementary Figure S1. An example of the extracted solar response κ (in the unit of °C/W m^{-2}; red), the correlation coefficient ρ (blue) with $TSI(t)$, and the separation parameter γ (yellow) as a function of the truncation parameter of the SVD pre-processing. The surface air temperature used in this example is HadCRUT5.

a few values of r. κ reaches a plateau of 0.0837°C/W m^{-2} when r is 30. Meanwhile, ρ also reaches a plateau of 0.83. In contrast, γ keeps increasing after r is 37 and it increases mostly abruptly when r is greater than 50 (increasing from 20 when r =52 to 90 when r = 57). Such an abrupt increase in γ near the maximum possible value of r is due to the null space and therefore should be avoided.

We performed the above analysis for the GISTEMP, NOAA, and ERA observational data and the CMIP6 model historical simulations. We found that $r = 30$ is generally applicable to all datasets. Thus, the results presented in the main text are derived using $r = 30$.

We assess the maximum uncertainty due to the choice of r by comparing with another approach adopted by Camp and Tung[4], who determined r by finding the maximum change in the rate of change of γ with respect to r. Their method would have chosen $r = 38$ in the case of HadCRUT5, based on the yellow curve in Supplementary Figure S1, whence $\kappa = 0.0827$°C/W m^{-2}, which is 1.19% lower than that at $r = 30$. We repeat the calculation of κ from the 200 ensemble members of HadCRUT5 using $r = 30$ and 38. The resulting distribution of κ has a median value 0.7% lower than those obtained using $r = 30$. Thus, we conclude that the uncertainty due to the choice of r is of order 1%.

Supplementary Section S2. Initialization configurations of CMIP6 models

In the main text, we selected a subset of CMIP6 models with sufficiently long spin-up and pre-industrial control simulations to ensure that the surface temperature in the models have equilibrated before the solar responses are simulated. Supplementary Table S1 lists the details of the initialization configurations of the CMIP6 models. The design of the CMIP6 experiments is described in Pascoe et al.[5] In one of our previous works using the GISS model[6], we showed that the ocean may take a few thousand years to reach the equilibrium state. However, only a few CMIP6 models have such long spin-up and pre-industrial control simulations. In the main text, we selected a subset of models whose (1) the spin-up and pre-industrial control times are well documented and (2) the combined spin-up and pre-industrial control simulations are at least 400 years before branching off to the historical run.

Supplementary Table S1: The numbers of historical runs available (#hruns), the equilibrium climate sensitivities (ECS)[7], the transient climate responses (TCR)[7], radiative forcing of CO_2 doubling[8], the solar responses (κ), duration of the spin-ups, duration of the pre-industrial control runs (piCtrl), and the branch-off times of the spin-ups from which the pre-industrial control runs start. The details of this information can be found from the references cited. Highlighted in orange are the models used in Figures 4 and 5 of the main text. Highlighted in pink are models not used in this study because of inadequate spin-up or preindustrial control of their oceans.

Model	#hruns	ECS (°C)	TCR (°C)	$F_{2\times CO2}$ (W m^{-2})	κ (°C /W m^{-2})	Spin-up (years)	piCtrl (years)	Branch-off time (years)	Ref #
ACCESS-CM2	3	4.7	2.1	--	0.093	950	500	0, 50, 100	9
ACCESS-ESM1-5	10	3.9	2	--	0.065	100	900	60, 80, ..., 240	--
AWI-CM-1-1-MR	5	3.2	2	--	0.073	500	500	150, 175, ..., 250	10
AWI-ESM-1-1-LR	1	--	--	--	0.056	1692[a] (land)<br>60 (ocean)	360[a]	260[a]	11
BCC-CSM2-MR	4	3	1.7	3.1	0.089	300	600	340	12
BCC-ESM1	3	3.3	1.8	3.01	0.084	400	450	245, 250, 255	13
CAMS-CSM1-0	4	2.3	1.7	4.17	0.051	20 (land)<br>50 (coupled)<br>(Start from resting ocean)	500	350	14<br>15
CANESM5	65	5.6	2.7	3.68	0.085	3500	800	0, 50, ..., 1200	16
CANESM5-CANOE	3	--	--	--	0.086	3500	500	0, 50, 150	16
CAS-ESM2-0	4	--	--	--	0.022	--	300	300	17
CESM2	11	5.2	2	3.26	0.027	133[a]	1200	501–871	18

CESM2-FV2	3	--	--	--	0.010	--	500	30	--
CESM2-WACCM	3	4.8	2	3.3	0.046	133[a]	500	56–71	[18]
CESM2-WACCM-FV2	3	--	--	--	0.072	--	500	30	--
CIESM	3	--	--	--	0.079	300	500	200, 300, 400	[19]
CMCC-CM2-SR5	1	--	--	--	0.095	1320 (land)	250	0	[20]
CNRM-CM6-1	30	4.8	2.1	3.64	0.067	750[a]	1000	0–800	[21]
CNRM-CM6-1-HR	1	4.3	2.5	3.96	0.008	300[a]	300	0	--
CNRM-ESM2-1	10	4.8	1.9	2.97	0.054	1500 (ocean)<br>100[a]	500	0–500	[22]
E3SM-1-0	5	5.3	3	3.33	0.097	400<br>50 (high res)	500	100, 150, ..., 300	[23]<br>[24]
E3SM-1-1	1	--	--	--	0.079	Start from E3SM1-0's spin-up	164	0	[25]
E3SM-1-1-ECA	1	--	--	--	0.065	Start from E3SM1-0's spin-up	164	0	--
EC-EARTH3	15	4.3	--	3.31	0.080	2000 (OMIP)	--	0	[26]
EC-EARTH3-LR	--	--	--	--	--	--	200	--	--
EC-EARTH3-VEG	3	4.3	2.6	3.37	0.103	180[a]	297	0, 100, 180	--
EC-EARTH3-VEG-LR	3	--	--	--	0.096	450[a]	500	0, 100, 200	--
FGOALS-F3-L	3	3	2.1	4.17	0.055	34[a]	1160	600	[27]
FGOALS-G3	6	--	--	--	0.090	--	700	170	--
FIO-ESM-2-0	3	--	--	--	0.098	300	700	0, 30, 50	[28]
GFDL-CM4	1	3.9	2.1	3.19	0.070	> 600	650	100, 150, 200	[29,30]
GFDL-ESM4	3	2.6	1.6	--	0.103	~few 100s	900	100, 150, 200	[31]
GISS-E2-1-G	32	2.7	1.8	3.94	0.072	> 500	1000	--	[32]
GISS-E2-1-G-CC	1	--	--	--	0.045	Should be same as above	--	--	[32]
GISS-E2-1-H	23	3.1	1.9	3.53	0.061	Should be same as above	--	--	[32]
GISS-E2-2-G	0	2.4	1.7	--	--	Should be same as above	--	--	[32]

HADGEM3-GC31-LL	4	5.6	2.6	3.49	0.066	615	500	0–100	33,34
HADGEM3-GC31-MM	2	5.4	2.6	--	0.109	224	500	0–30	33,34
IITM-ESM	0	--	1.7	--	--	300	500	--	35
INM-CM4-8	1	1.8	1.3	2.7	0.092	--	530	--	36
INM-CM5-0	0	1.9	--	--	--	--	1200	--	36
IPSL-CM6A-LR	32	4.6	2.3	3.41	0.070	~few 100s and 100[a] (from 1750 to 1850)	2000 (i1) 250 (i2)	20, 40, ..., 640	37
KACE-1-0-G	3	4.5	1.4	--	0.039	60	--	--	38
MCM-UA-1-0	2	3.7	1.9	--	0.030	--	500	--	--
MIROC-ES2L	10	2.7	1.6	4.11	0.041	3000 (ocean)<br>30,000 (land)<br>350 (coupled)	500	0, 10, ...	39
MIROC6	50	2.6	1.6	3.65	0.089	1000 (ocean)	800	0, 30, ..., 750	40
MPI-ESM1-2-HR	10	3	1.7	3.65	0.063	150 or 1000[a]	500	--	41<br>42
MPI-ESM1-2-LR	10	3	1.8	4.10[43]	0.045	12,000	1000 (r1) 100 (r2)	--	43
MRI-ESM2-0	6	3.2	1.6	3.43	0.069	1000	700	0, 50, ..., 200	44
NESM3	5	4.7	2.7	3.62	0.114	2000 (ocean)<br>400 (coupled)	500	--	45
NORCPM1	30	--	1.6	--	0.043	--	--	--	46
NORESM2-LM	30	2.5	1.5	3.44	0.130	1400	--	--	47
SAM0-UNICON	1	3.7	2.3	3.89	0.068	54[a]	700	250	48
TAIESM1	1	--	--	--	0.042	200[a]	500	270	--
UKESM1-0-LL	17	5.3	2.8	3.61	0.089	5000 (ocean)<br>1000 (land)<br>500 (coupled)<br>110[a]	1100	--	49

[a] Based on the global attributes "parent time" in the NetCDF files archived in CMIP6 database.

Supplementary References

1 Fisher, R. A. The use of multiple measurements in taxonomic problems. Ann. Eugen. 7, 179–188, doi:10.1111/j.1469-1809.1936.tb02137.x (1936).

2 Ripley, B. D. Pattern recognition and neural networks. (Cambridge University Press, 1996).

3 Schneider, T. & Held, I. M. Discriminants of twentieth-century changes in Earth surface temperatures. J. Clim. 14, 249–254, doi:10.1175/1520-0442(2001)014<0249:LDOTCC>2.0.CO;2 (2001).

4 Camp, C. D. & Tung, K.-K. The influence of the solar cycle and QBO on the late-winter stratospheric polar vortex. J. Atmos. Sci. 64, 1267–1283, doi:10.1175/JAS3883.1 (2007).

5 Pascoe, C., Lawrence, B. N., Guilyardi, E., Juckes, M. & Taylor, K. E. Documenting numerical experiments in support of the Coupled Model Intercomparison Project Phase 6 (CMIP6). Geosci. Model Dev. 13, 2149–2167, doi:10.5194/gmd-13-2149-2020 (2020).

6 Liang, M.-C., Lin, L.-C., Tung, K.-K., Yung, Y. L. & Sun, S. Transient Climate Response in Coupled Atmospheric-Ocean General Circulation Models. J. Atmos. Sci. 70, 1291–1296, doi:10.1175/JAS-D-12-0338.1 (2013).

7 Meehl, G. A. et al. Context for interpreting equilibrium climate sensitivity and transient climate response from the CMIP6 Earth system models. Science Adv. 6, eaba1981, doi:10.1126/sciadv.aba1981 (2020).

8 Zelinka, M. D. et al. Causes of Higher Climate Sensitivity in CMIP6 Models. Geophys. Res. Lett. 47, e2019GL085782, doi:10.1029/2019GL085782 (2020).

9 ACCESS official website. https://accessdev.nci.org.au/trac/wiki/access/ACCESS-CM2.

10 Semmler, T. et al. Simulations for CMIP6 With the AWI Climate Model AWI-CM-1-1. J. Adv. Model. Earth Syst. 12, e2019MS002009, doi:10.1029/2019MS002009 (2020).

11 Sidorenko, D. et al. Towards multi-resolution global climate modeling with ECHAM6-FESOM. Part I: model formulation and mean climate. Clim. Dyn. 44, 757-780, doi:10.1007/s00382-014-2290-6 (2015).

12 Wu, T. et al. The Beijing Climate Center Climate System Model (BCC-CSM): the main progress from CMIP5 to CMIP6. Geosci. Model Dev. 12, 1573–1600, doi:10.5194/gmd-12-1573-2019 (2019).

13 Wu, T. et al. Beijing Climate Center Earth System Model version 1 (BCC-ESM1): Model description and evaluation of aerosol simulations. Geosci. Model Dev. 13, 977–1005, doi:10.5194/gmd-13-977-2020 (2020).

14 Rong, X. et al. The CAMS climate system model and a basic evaluation of its climatology and climate variability simulation. J. Meteorol. Res. 32, 839–861, doi:10.1007/s13351-018-8058-x (2018).

15 Nan, S., Yang, J., Bao, Y., Li, J. & Rong, X. Simulation of the northern and southern hemisphere annular modes by CAMS-CSM. J. Meteorol. Res. 33, 934–948, doi:10.1007/s13351-019-8099-9 (2019).

16 Swart, N. C. et al. The Canadian Earth System Model version 5 (CanESM5.0.3). Geo. Model Dev. 12, 4823–4873, doi:10.5194/gmd-12-4823-2019 (2019).

17 Jin, J. B. et al. CAS-ESM2.0 Model Datasets for the CMIP6 Flux-Anomaly-Forced Model Intercomparison Project (FAFMIP). Adv. Atmos. Sci. 38, 296–306, doi:10.1007/s00376-020-0188-2 (2021).

18 Danabasoglu, G. et al. The Community Earth System Model Version 2 (CESM2). J. Adv. Model. Earth Syst. 12, e2019MS001916, doi:10.1029/2019MS001916 (2020).

19 Lin, Y. et al. Community Integrated Earth System Model (CIESM): Description and evaluation. J. Adv. Model. Earth Syst. 12, e2019MS002036, doi:10.1029/2019MS002036 (2020).
20 Cherchi, A. et al. Global Mean Climate and Main Patterns of Variability in the CMCC-CM2 Coupled Model. J. Adv. Model Earth System 11, 185-209, doi:10.1029/2018MS001369 (2019).
21 Voldoire, A. et al. Evaluation of CMIP6 DECK Experiments With CNRM-CM6-1. J. Adv. Model. Earth Syst. 11, 2177–2213, doi:10.1029/2019MS001683 (2019).
22 Séférian, R. et al. Evaluation of CNRM Earth System Model, CNRM-ESM2-1: Role of Earth system processes in present-day and future climate. J. Adv. Model. Earth Syst. 11, 4182−4227, doi:10.1029/2019MS001791 (2019).
23 Golaz, J. C. et al. The DOE E3SM Coupled Model version 1: Overview and evaluation at standard resolution. J. Adv. Model. Earth Syst. 11, 2089–2129, doi:10.1029/2018MS001603 (2019).
24 Caldwell, P. M. et al. The DOE E3SM Coupled Model version 1: Description and results at high resolution. J. Adv. Model. Earth Syst. 11, 4095–4146, doi:10.1029/2019MS001870 (2019).
25 E3SM-1-1. https://e3sm.org/data/get-e3sm-data/released-e3sm-data/v1-1-deg-data-cmip6/.
26 Wyser, K. et al. On the increased climate sensitivity in the EC-Earth model from CMIP5 to CMIP6. Geosci. Model Dev. 13, 3465–3474, doi:10.5194/gmd-13-3465-2020 (2020).
27 He, B. et al. CAS FGOALS-f3-L model dataset descriptions for CMIP6 DECK experiments. Atmos. Oceanic Sci. Lett. 13, 582−588, doi:10.1080/16742834.2020.1778419 (2020).
28 Bao, Y., Song, Z. & Qiao, F. FIO-ESM Version 2.0: Model description and evaluation. J. Geophys. Res. Oceans 125, e2019JC016036, doi:10.1029/2019JC016036 (2020).
29 Held, I. M. et al. Structure and Performance of GFDL's CM4.0 Climate Model. J. Adv. Model. Earth Syst. 11, 3691–3727, doi:10.1029/2019MS001829 (2019).
30 Winton, M. et al. Climate Sensitivity of GFDL's CM4.0. J. Adv. Model Earth Syst. 12, e2019MS001838, doi:10.1029/2019MS001838 (2020).
31 Dunne, J. P. et al. The GFDL Earth System Model Version 4.1 (GFDL-ESM 4.1): Overall coupled model description and simulation characteristics. J. Adv. Model Earth Systems 12, doi:10.1029/2019MS002015 (2020).
32 Kelley, M. et al. GISS-E2.1: Configurations and Climatology. J. Adv. Model. Earth Syst. 12, e2019MS002025, doi:10.1029/2019MS002025 (2020).
33 Kuhlbrodt, T. & Jones, C. Starting the CMIP6 simulations with HadGEM3 GC3.1. UKESM December Newsletter, https://ukesm.ac.uk/portfolio-item/starting-cmip6-simulations-hadgem3-gc3-1/ (2017).
34 Kuhlbrodt, T. et al. The low-resolution version of HadGEM3 GC3.1: Development and evaluation for global climate. J. Adv. Model. Earth Syst. 10, 2865–2888, doi:10.1029/2018MS001370 (2018).
35 Swapna, P. et al. Long-term climate simulations using the IITM Earth System Model (IITM-ESMv2) with focus on the South Asian monsoon. J. Adv. Model. Earth Syst. 10, 1127–1149, doi:10.1029/2017MS001262 (2018).
36 Volodin, E. & Gritsun, A. Simulation of observed climate changes in 1850–2014 with climate model INM-CM5. Earth Syst. Dyn. 9, 1235–1242, doi:10.5194/esd-9-1235-2018 (2018).
37 Boucher, O. et al. Presentation and Evaluation of the IPSL-CM6A-LR Climate Model. J. Adv. Model. Earth Syst. 12, e2019MS002010, doi:10.1029/2019MS002010 (2020).

38 Lee, J. et al. Evaluation of the Korea Meteorological Administration Advanced Community Earth-System model (K-ACE). Asia-Pacific J. Atmos. Sci. 56, 381–395, doi:10.1007/s13143-019-00144-7 (2020).
39 Hajima, T. et al. Development of the MIROC-ES2L Earth system model and the evaluation of biogeochemical processes and feedbacks. Geosci. Model Dev. 13, 2197–2244, doi:10.5194/gmd-13-2197-2020 (2020).
40 Tatebe, H. et al. Description and basic evaluation of simulated mean state, internal variability, and climate sensitivity in MIROC6. Geosci. Model Dev. 12, 2727–2765, doi:10.5194/gmd-12-2727-2019 (2019).
41 Gutjahr, O. et al. Max Planck Institute Earth System Model (MPI-ESM1.2) for the High-Resolution Model Intercomparison Project (HighResMIP). Geosci. Model Dev. 12, 3241–3281, doi:10.5194/gmd-12-3241-2019 (2019).
42 Haarsma, R. J. et al. High Resolution Model Intercomparison Project (HighResMIP v1.0) for CMIP6. Geosci. Model Dev. 9, 4185–4208, doi:10.5194/gmd-9-4185-2016 (2016).
43 Mauritsen, T. et al. Developments in the MPI-M Earth System Model version 1.2 (MPI-ESM1.2) and its response to increasing CO2. J. Adv. Model. Earth Syst. 11, 998–1038, doi:10.1029/2018MS001400 (2019).
44 Yukimoto, S. et al. The Meteorological Research Institute Earth System Model version 2.0, MRI-ESM2.0: Description and basic evaluation of the physical component. J. Meteorol. Soc. Jpn. 97, 931–965, doi:10.2151/jmsj.2019-051 (2019).
45 Cao, J. et al. The NUIST Earth System Model (NESM) version 3: Description and preliminary evaluation. Geosci. Model Dev. 11, 2975–2993, doi:10.5194/gmd-11-2975-2018 (2018).
46 Jackson, L. C. et al. The mean state and variability of the North Atlantic circulation: A perspective from ocean reanalyses. J. Geophys. Res. Oceans 124, 9141–9170, doi:10.1029/2019JC015210 (2019).
47 Seland, Ø. et al. Overview of the Norwegian Earth System Model (NorESM2) and key climate response of CMIP6 DECK, historical, and scenario simulations. Geosci. Model Dev. 13, 6165–6200, doi:10.5194/gmd-13-6165-2020 (2020).
48 Park, S., Shin, J., Kim, S., Oh, E. & Kim, Y. Global Climate Simulated by the Seoul National University Atmosphere Model Version 0 with a Unified Convection Scheme (SAM0-UNICON). J. Clim. 32, 2917–2949, doi:10.1175/JCLI-D-18-0796.1 (2019).
49 Yool, A. et al. Spin-up of UK Earth System Model 1 (UKESM1) for CMIP6. J. Adv. Model. Earth Syst. 12, e2019MS001933, doi:10.1029/2019MS001933 (2020).
